# Rift Valley fever virus detection in susceptible hosts with special emphasis in insects

**DOI:** 10.1038/s41598-021-89226-z

**Published:** 2021-05-10

**Authors:** K. M. Gregor, L. M. Michaely, B. Gutjahr, M. Rissmann, M. Keller, S. Dornbusch, F. Naccache, K. Schön, S. Jansen, A. Heitmann, R. König, B. Brennan, R. M. Elliott, S. Becker, M. Eiden, I. Spitzbarth, W. Baumgärtner, C. Puff, R. Ulrich, M. H. Groschup

**Affiliations:** 1 Department of Pathology, University of Veterinary Medicine Hannover, Hannover, Germany; 2 Institute of Novel and Emerging Infectious Diseases, Friedrich-Loeffler-Institute, Greifswald, Germany; 3 Institute for Parasitology and Research Center for Emerging Infections and Zoonoses, University of Veterinary Medicine Hannover, Hannover, Germany; 4 Department of Arbovirology, Bernhard Nocht Institute for Tropical Medicine, Hamburg, Germany; 5 MRC-University of Glasgow Centre for Virus Research, Glasgow, Scotland, UK; 6 Institute of Veterinary Pathology, Faculty of Veterinary Medicine, Leipzig University, Leipzig, Germany

**Keywords:** Biological techniques, Biomarkers, Diseases, Medical research, Pathogenesis

## Abstract

Rift Valley fever phlebovirus (RVFV, *Phenuiviridae*) is an emerging arbovirus that can cause potentially fatal disease in many host species including ruminants and humans. Thus, tools to detect this pathogen within tissue samples from routine diagnostic investigations or for research purposes are of major interest. This study compares the immunohistological usefulness of several mono- and polyclonal antibodies against RVFV epitopes in tissue samples derived from natural hosts of epidemiologic importance (sheep), potentially virus transmitting insect species (*Culex quinquefasciatus, Aedes aegypti*) as well as scientific infection models (mouse, *Drosophila melanogaster,* C6/36 cell pellet). While the nucleoprotein was the epitope most prominently detected in mammal and mosquito tissue samples, fruit fly tissues showed expression of glycoproteins only. Antibodies against non-structural proteins exhibited single cell reactions in salivary glands of mosquitoes and the C6/36 cell pellet. However, as single antibodies exhibited a cross reactivity of varying degree in non-infected specimens, a careful interpretation of positive reactions and consideration of adequate controls remains of critical importance. The results suggest that primary antibodies directed against viral nucleoproteins and glycoproteins can facilitate RVFV detection in mammals and insects, respectively, and therefore will allow RVFV detection for diagnostic and research purposes.

## Introduction

Rift Valley fever phlebovirus (*Phenuiviridae*, RVFV) is a zoonotic, emerging and vector-borne disease, which was firstly discovered in sheep in the Rift Valley province in Kenya, Africa^1,2^. To date it is endemic in Africa^3,4^ and the Arabian Peninsula^5^, where outbreaks depend on climatic and ecological conditions, vector behavioral factors^3,4,6^ and viral genetic diversity^7–9^. This arthropod-borne disease affects humans as well as domestic animals, including ruminants and camels, which serve as amplifying hosts^10–12^.

The majority of human infections leads to febrile disease, while small percentages result in hemorrhagic fever, maculo-retinitis, late-onset encephalitis, miscarriage as well as hepatic and renal failure^10,13–15^. Ruminants, especially sheep and goats, suffer from sudden and widespread abortions and high mortality rates in neonates and juveniles^16,17^. Humans usually acquire infections by contact with infected animal tissues and fluids, but animals are usually infected by mosquito bite^12,18–26^.

Over 50 mosquito species have tested positive for RVFV^4^, including *Culex pipiens quinquefasciatus* (*Cx.qu.*)^27–31^ and *Aedes aegypti* (*A.ae.*)^28,29,32^ as natural vectors in endemic areas and *Aedes albopictus* (*A.alb.*)^33,34^ as a potential vector in Europe and the United States. The abundance of competent vectors in Africa^4,23^ along with wind transfer of mosquitoes^35^, wildlife fluctuation^36^, irrigation farming^29^ and suitable climatic conditions^37^ promote the re-emergence of RVFV in Africa. Furthermore, RVFV poses an imminent threat to non-African regions including Europe and North America^23,38–40^ as livestock trading^18,25,41^, globalization^42^ and changing climatic conditions^43,44^ promote the emergence of this disease. Another important issue comprises that various native mosquitoes on the European continent present as potent vectors of RVFV in several studies^34,45,46^. Therefore, RVFV is of major concern to public health and of economic importance. In addition, it represents a prime example of a disease, which is currently approached by one-health concepts^4,47^.

Given this background, current investigations focus on molecular pathogenesis during the course of disease within mammalian and insect hosts. RVFV is a single-stranded, negative- or ambisense RNA virus and consists of three RNA segments (S, M, and L). The L segment encodes the RNA dependent polymerase, responsible for virus transcription, while the M segment encodes non-structural proteins (78 kDa NSm, 14 kDa NSm) and two glycoproteins (Gc, Gn)^48,49^. The S segment encodes for the nucleoprotein (N) and a non-structural protein (NSs)^12,50^. The glycoproteins enable the fusion of virions with host cells, while the nucleoprotein encapsidates the genome. NSs is regarded to be the main virulence factor and its mechanism of action facilitates the evasion of the mammalian innate immune system^50–52^. However, NSs might not be required for RVFV maintenance in mosquitoes^53,54^. While the 14 kDa NSm protein is able to suppress caspase-induced apoptosis in mammalian host cells supporting viral pathogenesis^55,56^, the 78 kDa Nsm and the 14 kDa Nsm are discussed to be of minor importance for virus maturation and replication in mammals^48,55,57^. In contrast, the 78 kDa Nsm protein plays an important role in insects^58,59^.

Beside natural hosts^60–63^, laboratory animals such as mice, rats, gerbils, but also monkeys are widely used models of experimental RVFV infection in order to assess mechanisms of disease, potential vaccines, or therapeutics^51,64,65^. In addition, fruit flies serve as model organisms to understand several aspects of disease in mammals and insects, including innate immune pathways^66–68^ and viral pathogenesis^69,70^. Especially the mode of neuroinvasion in mammals or the influence on nervous functions in mosquitoes are not fully elucidated in RVFV pathogenesis so far^71–75^. Therefore, fruit flies enable targeted investigations, since many physiological and biochemical mechanisms are highly conserved^76,77^.

Veterinary and public health authorities as well as scientific investigations on RVFV require diagnostic tools for virus detection. In this context, visualization of viral proteins within tissue samples is essential to identify its cell tropism and is commonly assessed by using immunohistochemistry. To facilitate these investigations the aim of the present study was to analyze comparatively several mono- as well as polyclonal antibodies directed against RVFV.

This study included different species of interest in RVFV research. In accordance with the natural spread, sheep and RVFV transmitting mosquitoes such as *Cx.qu.* and *A.ae.* were used to assess the diagnostic value of RVFV targeting antibodies in immunohistochemistry. Furthermore, mice, fruit flies (*Drosophila melanogaster*) and an insect cell line (C6/36) of *A.alb* were evaluated as models of infection.

## Results

This study analyzed the usability of various monoclonal and polyclonal antibodies directed against RVFV (Table 1) using immunohistochemistry on tissues from sheep and RVFV transmitting mosquitoes (*C.qu*., *A.ae*.). In addition, mice, *Drosophila melanogaster* strain *cinnabar brown* (*D.mel. cnbw*) and strain *yellow-white* (*D.mel. yw*) as well as an insect cell line (C6/36) of *A.alb.* were also evaluated as models of infection.

**Table 1:** List of antibodies tested to detect RVFV.

Primary antibody	Epitope	Clonality/host species	Dilution	Pretreatment	Secondary antibody	Chromogen	Source
Np9	Nucleoprotein	mc, mouse	1:200	Citrate buffer*	Goat anti-mouse	DAB	FLI^81^
polyNp	Nucleoprotein	pc, rabbit	1: 3000	None	Goat anti-rabbit	DAB	FLI^81^
S24Np	Nucleoprotein	pc, sheep	1:8000 (mammals)/ 1:130.000 (insects)	None	Rabbit anti-sheep	DAB	FLI^82^
Gc9A9	Glycoprotein Gc	mc, mouse	1:200	Citrate buffer*	Goat anti-mouse	DAB	FLI^81^
polyGc	Glycoprotein Gc	pc, rabbit	1:3000	None	Goat anti-rabbit	DAB	FLI^81^
Gn164b	Glycoprotein Gn	mc, mouse	1:100	Citrate buffer*	Goat anti-mouse	DAB	FLI^81^
7B6	Glycoprotein Gn	mc, mouse	1:50	Citrate buffer*	Goat anti-mouse	DAB	USAMRIID^83^
polyGn	Glycoprotein Gn	pc, rabbit	1:3000	None	Goat anti-rabbit	DAB	FLI^84^
NSs5F12	Ns protein NSs	mc, mouse	1:100	Citrate buffer*	Goat anti-mouse	DAB	FLI^85^
NSm1E9A2	Ns protein NSm	mc, mouse	1:50	Citrate buffer*	Goat anti-mouse	DAB	FLI^85^

*: Microwaved in citrate buffer for 20 min at 600 W; DAB: 3,3′-diaminobenzidine; FLI: Friedrich–Loeffler-Institute, Greifswald; mc: monoclonal; Ns: non-structural, pc: polyclonal; USAMRIID: United States Army Medical Research Institute of Infectious Diseases, Fort Detrick, Maryland, USA.

### RT-qPCR

Initially, liver tissues of sheep and mice as well as homogenates of fruit flies and mosquitoes were investigated using RT-qPCR for verification of RVFV-infection.

Brain homogenates of five infected *D.mel. cnbw* and *yw* each revealed Ct values of 27.15–32.89 (*D.mel*. *cnbw*) and 26.55–31.27 (*D.mel. yw*). RT-qPCR with formalin-fixed and paraffin-embedded (FFPE) material of RVFV-infected and mock-infected mosquito (*Cx.qu*. and *A.ae.*) as well as fruit fly (*D.mel. cnbw* and *yw*) species lacked any amplifiable genetic material.

Liver homogenates of RVFV (strain 35/74) infected sheep revealed Ct values ranging from 25.22 to 27.94 indicating the presence of viral genome in the organ. Crl:NU(NCr)-*Foxn1*^*nu*^ mice infected with the highly virulent RVFV strain 35/74 were strongly positive (Ct values ranging from 12.84 to 13.13). C57Bl/6-IFNAR^tmAgt^ mice infected with RVFV (strain MP12) were positive (Ct values ranging from 16.33 to 19.57). Samples of mock-infected sheep and from both mock-infected mouse strains were negative for RVFV-specific nucleic acids.

### Antigen intensity and dissemination

Immunoreactivity of the different antibodies was characterized in RVFV-infected insects and mammals. A detailed overview about the organ involvement is given in Table 2.

**Table 2:** Immunopositive signals in RVFV-infected specimens.

Antibody	Insects					Mammals		
	*C6/36 pellet*	*Cx.qu.*	*A.ae.*	*D.mel. cnbw*	*D.mel. yw*	Sheep	mice C57Bl/6-IFNAR^tmAgt^	mice Crl:NU(NCr)-*Foxn1*^*nu*^
Np9	++^#^	+*^,#^	+*^,#^	+*^,#^	+*^,#^	+++*^,#^	+++^#^	+++^#^
polyNp	++^#^	n.e.	n.e.	n.e.	n.e.	++^#^	+++^#^	−
S24Np	++^#^	+*^,#^	+*^,#^	n.e.	n.e.	+++*^,#^	+++	+++
Gc9A9	++^#^	+*^,#^	+*^,#^	+*^,#^	+*^,#^	++	+++*^,#^	++^#^
polyGc	+^#^	n.e.	n.e.	n.e.	n.e.	+^#^	++	+++
Gn164b	+	+^#^	+*^,#^	−*	−*	+^#^	++^#^	++^#^
7B6	+	+^#^	+*^,#^	+*	−*	−^#^	−	++*^,#^
polyGn	+^#^	n.e.	n.e.	n.e.	n.e.	+^#^	+^#^	+^#^
NSs5F12	+	+*^,#^	+*^,#^	−^#^	−^#^	−^#^	++^#^	++*^,#^
NSm1E9A2	−	+	+	−^#^	−^#^	−^#^	−^#^	++*^,#^

*A.ae.: Aedes aegypti; Cx.qu.: Culex pipiens quinquefasciatus; D.mel. cnbw: Drosophila melanogaster cinnabar brown; D.mel. yw: Drosophila melanogaster yellow-white;* n.a.: not assessed; n.e.: not evaluable; +: positive reaction in insect tissue, low numbers (< 30%) of positive cells in mammals and C6/36 cell pellet; ++: moderate numbers (30–60%) of positive cells in mammals and C6/36 cell pellet; +++: high numbers (> 60%) of positive cells in mammals and C6/36 cell pellet; −: no reaction; *: false positive labeling in non-infected specimens; #: unspecific background of varying degree.

#### C6/36 cell pellet

**Figure 1–15:**
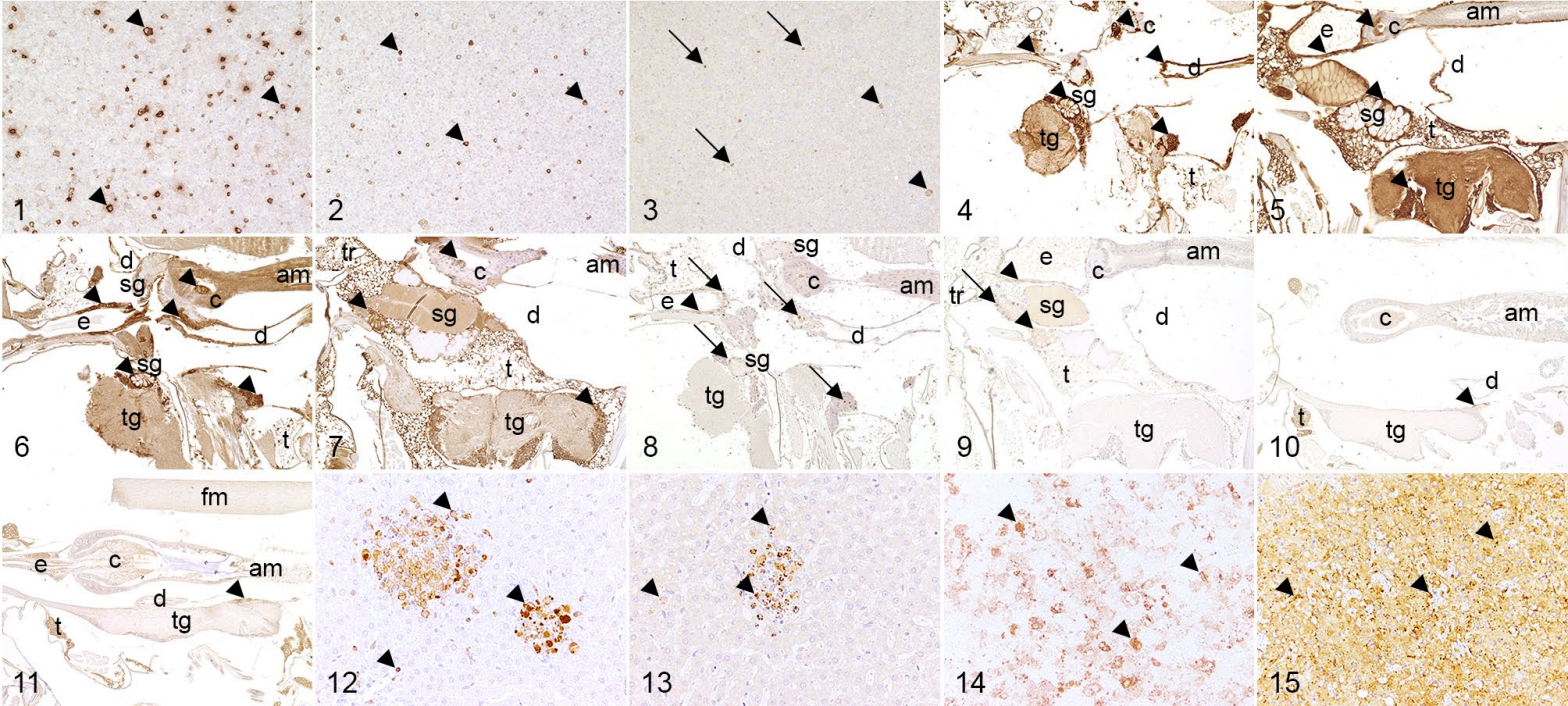
Comparison of epitope expression in mammal and insect specimens with intracytoplasmic (arrowheads) or intranuclear (arrows), granular signals for Rift Valley fever virus (RVFV). Figure 1–3: Immunoreactivity in RVFV-infected C6/36 cell pellet for the antibodies Np9 (1), Gc9A9 (2) and NSs5F12 (3). Figure 4–9: Immunohistochemical demonstration of the antibodies Np9 (4-5), Gc9A9 (6-7) and NSs5F12 (8-9) in RVFV-infected *Culex pipiens quinquefasciatus* (Fig. 4, 6, 8) and *Aedes aegypti* (Fig. 5, 7, 9). Figure 10: Immunoreactivity in RVFV-infected *Drosophila melanogaster* *yellow-white* for the antibody Np9. Figure 11: Immunohistochemical demonstration of the antibody Gc9A9 in *Drosophila melanogaster cinnabar brown*. Figure 12–13: Immunoreactivity in RVFV-infected ovine liver tissue for the antibodies Np9 (12) and Gc9A9 (13). Figure 14–15: Immunohistochemical demonstration of the antibodies S24Np (14) and Gc9A9 (15) in RVFV-infected C57Bl/6-IFNAR^tmAgt^ murine liver tissue. Note the signal within multifocal lesions in ovine liver samples (Fig. 12–13). In contrast, C57Bl/6-IFNAR^tmAgt^ mice exhibited a diffuse expression of Rift Valley fever antigen (Figure 14–15). *am* anterior midgut, *c* cardia, *d* diverticulum, *e* esophagus, *fm* flight muscle, *sg* salivary gland, *tg* thoracic ganglia, *t* trophocytes.

Nucleoprotein was present as a cytoplasmic, granular signal within moderate numbers (30–60%) of RVFV-infected C6/36-cells using the antibodies Np9 (Fig. 1), polyNp and S24Np (Table 2). The use of the antibody Gc9A9 yielded moderate numbers (30–60%) of RVFV-infected C6/36 cells with the same reaction pattern as seen for anti-nucleoprotein antibodies (Fig. 2). All remaining antibodies directed against glycoproteins (polyGc, Gn146b, 7B6 and polyGn) labeled low numbers (< 30%) of RVFV-infected C6/36 cells with a cytoplasmic, granular reaction. Antigen was present in low numbers of RVFV-infected cells (< 30%) with an intracytoplasmic and nuclear, granular reaction using NSs5F12 (Fig. 3), while NSm1E9A2 did not show a reliable reaction in virus infected C6/36 cells. Non-infected cells presented a low to moderate background for Np9, Gc9A9 and all polyclonal antibodies. In contrast, non-infected controls were free of labeling as well as background staining using remaining monoclonal antibodies (see Supplementary Fig. S1-S22 online).

#### Mosquito species

In both RVFV-infected mosquito species, a cytoplasmic immunoreactivity was present in various organs using antibodies directed against the nucleoprotein. While signals were strong and well defined with Np9 (Fig. 4–5), a more subtle, granular signal was observed using S24Np. Both aforementioned antibodies stained single cortical cells from head ganglia as well as the salivary gland falsely positive in mock-infected individuals. Moreover, a variable low to moderate background staining was present in both antibodies in infected and mock-infected specimens. The use of ovine normal serum as an antibody negative control revealed a variable low to high background staining in *Cx.qu*. and *A.ae*. Using the antibody polyNp, a distinction between a cytoplasmic, granular labeling from the variably intense background was not possible in infected as well as mock-infected individuals (Table 2).

Application of antibodies directed against glycoproteins led to a distinct, cytoplasmic, granular labeling of varying degree. Gc9A9 (Fig. 6–7) yielded the most intense signal and broadest organ involvement (Table 3) in infected mosquitos. A similar signal of decreasing amount was observed using Gn164b and 7B6. Mock-infected controls presented a false positive, cytoplasmic, homogenous, partly granular labeling of single cortical cell bodies of body ganglia, oenocytes, nephrocytes and salivary gland using Gc9A9. Furthermore, immunostaining with Gn164b and 7B6 revealed a false positive staining in oenocytes and/or a single cortical cell of the thoracic ganglion in mock-infected *A.ae.,* while they remained negative in mock-infected *Cx.qu*. In addition, infected and mock-infected mosquitos displayed a low to moderate background staining of trophocytes, gastrointestinal tract and ovaries with Gc9A9. In contrast, a background reaction was lacking or mild using Gn164b and 7B6.

**Table 3:** Organs with immunopositive signals in RVFV-infected insect specimens.

Antibody	Salivary gland				Cortical cell bodies of ganglia				Foregut/hindgut			
	*Cx.qu.*	*A.ae.*	*D.mel. cnbw*	*D.mel. yw*	*Cx.qu.*	*A.ae.*	*D.mel. cnbw*	*D.mel. yw*	*Cx.qu.*	*A.ae.*	*D.mel. cnbw*	*D.mel. yw*
Np9	+*	+*	n.a.	n.a.	+*	+*	+*	−*	+/+	+/+	−^#^	−^#^
polyNp	n.e.	n.e.	n.e.	n.e.	n.e.	n.e.	n.e.	n.e.	n.e	n.e.	n.e.	n.e.
S24Np	+*	+*	n.a.	n.a.	+*	+*	n.e	−^#^	+/−	+/+	n.e	−^#^
Gc9A9	+*	+*	n.a.	n.a.	+*	+*	+*	+*	+/+^#^	+/+^#^	−^#^	−^#^
polyGc	n.e.	n.e.	n.e.	n.e.	n.e.	n.e.	n.e.	n.e.	n.e	n.e.	n.e.	n.e.
Gn164b	+	+	n.a.	n.a.	+	+	−	−	+/+	+/+	−	−^#^
7B6	+	+	n.a.	n.a.	−	−*	+	−	+/−	+/+	−	−^#^
polyGn	n.e.	n.e.	n.e.	n.e.	n.e.	n.e.	n.e.	n.e.	n.e.	n.e.	n.e.	n.e.
NSs5F12	+	+	n.a.	n.a.	+	−	−	−	+/−	−	−^#^	−^#^
NSm1E9A2	+	+	n.a.	n.a.	−	−	−	−	−	+/−	−^#^	−^#^

*A.ae.: Aedes aegypti; Cx.qu.: Culex pipiens quinquefasciatus; D.mel. cnbw: Drosophila melanogaster cinnabar brown; D.mel. yw: Drosophila melanogaster yellow-white*; n.a.: not assessed; n.e.: not evaluable; +: positive reaction; −: no reaction. *false positive labeling in non-infected specimens. ^#^unspecific background of varying degree.

The polyclonal antibodies polyGc and polyGn showed a variably strong background reaction and a rather diffuse cytoplasmic labelling pattern in infected and mock-infected specimens resulting in the impossibility to identify a virus specific signal.

A mild, intracytoplasmic and nuclear, granular labeling was present using NSs5F12 in infected mosquitos (Fig. 8–9). Mock-infected controls displayed a mild background staining in variable cells such as oenocytes, trophocytes and nephrocytes. Labeling with NSm1E9A2 revealed a mild, cytoplasmic, granular reaction in RVFV-infected *Cx.qu.* and *A.ae.*, while mock-infected controls showed no reactivity (see Supplementary Fig. S23-S66 online).

#### Fruit flies

Both strains of fruit flies revealed the same staining pattern with most of the antibodies used. A cytoplasmic and granular labeling was present in RVFV-infected fruit flies using the antibody Np9 (Fig. 10), Table 2). Mock-infected controls presented a false positive, cytoplasmic, granular signal in cortical cell bodies of head ganglia and a variable, mild background staining of fat body and gastrointestinal tract. Due to the high background reaction, a virus-specific signal was not discernable using the antibodies polyNP and S24Np in infected and mock-infected individuals. Antibody negative controls treated with ovine normal serum revealed a variably strong background staining.

The antibody Gc9A9 presented the strongest and broadest cytoplasmic, granular immunolabeling (Fig. 11, Table 3) in RVFV-infected individuals. Using the antibodies Gn164b and 7B6 resulted in a diffuse, cytoplasmic immunoreactivity in tropho- and oenocytes, whereby the antibody 7B6 also labeled cytoplasm of cortical cell bodies of head ganglia in a granular pattern in *D.mel. cnbw*. However, immunostaining with Gc9A9 yielded a granular, false positive labeling of cortical cell bodies of head ganglia and a variably strong background reaction in various organs including the gastrointestinal tract and the fat body in mock-infected controls. Using the antibodies Gn164b and 7B6, mock-infected controls presented a mild, diffuse, cytoplasmic labeling of the fat body. As with mosquitoes, using the antibodies polyGn and polyGc, specific immunolabeling was indiscernible from a high background reaction.

Regarding non-structural proteins, labeling was diffuse in the cytoplasm of trophocytes and oenocytes in RVFV-infected fruit flies using the antibodies NSs5F12 and NSm1E9A2. In mock-infected individuals, a mild background reaction was present in fat body and gastrointestinal tract (see Supplementary Fig. S67-S110 online).

#### Sheep

In RVFV-infected sheep, the antibodies Np9 (Fig. 12) and S24Np showed a strong, cytoplasmic granular reaction within > 60% of hepatocytes and Kupffer cells. However, samples from mock-infected controls displayed a diffuse false positive reaction within Kupffer cells, while S24Np exhibited diffuse background labeling. PolyNp showed the same pattern with a lower intensity (30–60% of hepatocytes and Kupffer cells).

Gc9a9 (Fig. 13) displayed a moderate reactivity (30–60% of hepatocytes and Kupffer cells) within infected liver samples. PolyGn, Gn164b and PolyGc showed a mild (< 30% of hepatocytes and Kupffer cells) reaction in RVFV-infected samples and a diffuse, non-specific background labeling.

No immunolabeling with 7B6, NSs5F12 or NSm1E9A2 was observed in RVFV-infected sheep (see Supplementary Fig. S111-S132 online).

#### Mice

Immunolabeling of murine samples with Np9 and S24Np (Fig. 14) antibodies resulted in a granular, cytoplasmic signal in > 60% of hepatocytes and Kupffer cells with unspecific background staining by Np9. PolyNp exhibited no reactivity within Crl:NU(NCr)-*Foxn1*^*nu*^ mice, while in C57Bl/6-IFNAR^tmAgt^ mice the result equaled the Np9 reaction.

Gc9a9 (Fig. 15) and polyGc labeling was moderate (30–60% of hepatocytes and Kupffer cells) to strong (> 60% of hepatocytes and Kupffer cells) with mild unspecific background by Gc9a9 staining in both mouse strains.

Gn164b and polyGn labeling was mild (< 30% of hepatocytes and Kupffer cells) to moderate (30–60% of hepatocytes and Kupffer cells) with moderate background staining that was less pronounced in the samples of Crl:NU(NCr)-*Foxn1*^*nu*^ mice. 7B6 did not show any reaction in C57Bl/6-IFNAR^tmAgt^ mice, while it labeled moderate numbers (30–60%) of hepatocytes and Kupffer cells in infected and control samples from Crl:NU(NCr)-*Foxn1*^*nu*^ mice alike.

RVFV-infected murine liver samples displayed a moderate (30–60% of hepatocytes) cytoplasmic, granular labeling and moderate unspecific background staining using NSs5F12 while the reactivity of NSm1E9A2 was congruent with 7B6. Both antibodies against non-structural proteins exhibited false-positive reactions with mock-infected control samples of Crl:NU(NCr)-*Foxn1*^*nu*^ mice. (see Supplementary Fig. S133-S176 online).

## Discussion

The detection, control and surveillance of RVFV in vertebrate and invertebrate species is of increasing interest throughout the past decades as RVF poses a global public health and economic risk factor^23,38–40^. This study assessed the usefulness of a panel of 10 different antibodies to detect the presence of RVFV in FFPE tissue sections of mammal and insect specimens. Therefore, sheep as an economically important host, potentially RVFV transmitting mosquitoes (*Cx.qu., A.ae.*) and mice, fruit flies (*D.mel. cmnb* and *yw*) and a mosquito cell line (C6/36 cell line of *A.alb.*) as scientific models of infection were comparatively analyzed.

The detailed evaluation of obtained signals revealed, that the anti-nucleoprotein antibodies Np9 and S24Np yielded a strong immunopositive signal in all investigated specimens, except for S24Np in *D.mel.* In contrast, glycoprotein antibodies presented a reduced signal in RVFV-infected samples, except for Gc9A9 in insect specimens. In fruit flies, it produced the strongest antigen-positive signal. On the other hand, the application of 7B6 demonstrated no immunoreactivity in ovine tissue and *D.mel. yw*. The polyclonal antibodies directed against Np, Gn and Gc did not serve useful for detection of the RVFV proteins as they lacked any specific reaction. The non-structural protein antibodies failed to detect RVFV antigen conclusively in most FFPE tissue samples with the exception of mosquito specimens. While viral proteins were detectable by NSs5F12 in both mosquito species and C6/36 cells, the antibody NSm1E9A2 only produced a specific signal in *A.ae*. Noteworthy, even though RVFV MP12 strain shows multiple amino acid substitutions, distributed over all three segments, when comparing to its parental strain ZH548^78^, there was no notable difference in staining intensity among mice infected with RVFV 35/74 or RVFV MP12, respectively. Therefore, the known epitopes of monoclonal antibodies Gn164b, Gc9A9 (see Supplementary material online) and 7B6^79^, but also the unknown epitopes of Np9, NSs5F12 and NSm1E9A2 seem to be rather conserved.

The results in mammals are congruent with previous investigations indicating a prominent expression of the S segment in mammals over the M and L segment^80^. This is reflected in the reactivity of the applied antibodies. A study on antiviral RNAi in insects provides evidence that the M segment of RVFV is highly abundant in insects, which might explain the strong signal produced by the Gc9A9 antibody. Furthermore, the present study shows that detection of the nonstructural protein 78 kDa NSm was only possible in the salivary gland of mosquitos. Unfortunately, due to its small size fruit fly salivary glands were not present in all slides investigated. Therefore, no comparative data were obtained in this study regarding the 78 kDa NSm expression within the salivary gland of this species. The non-structural 78 kDa RVFV-NSm protein is important for RVFV infection in insects as studies with modified RVFV strains lacking the 78 kDa NSm and 14 kDa NSm proteins resulted in a reduction of virus infection, dissemination and transmission potential in *A.ae.* and to a lesser extent in *Cx.qu.*^81,82^.

In comparison to mice and sheep, an antigen-positive signal with the anti-NSs-antibody was detected in mosquitoes and C6/36 cells in this study to a higher extent. These findings are in disagreement with previous studies that show only low or no expression of NSs in insect cell lines when compared to mammalian cells^52,54^. Moreover, the cause of the unexpected observation that single cells in RVFV-infected mosquitoes and the infected C6/36 cell pellet display a nuclear and cytoplasmic immunoreactivity remains unclear and requires further studies. However, it is noteworthy that the observed intracellular and -nuclear distribution pattern of NSs might represent species-specific in vivo conditions of the RVFV infection cycle, especially as in vivo and in vitro observed mechanisms cannot simply be extrapolated across species or environmental settings in nature^83,84^.

Another observation that demands careful interpretation is the RVFV distribution and signal intensity in insect specimens. While RVFV antigens in fruit flies were mainly expressed in fat body and nervous system, they were present in almost the entire body in *Cx.qu.* and *A.ae.* mosquitoes^85^. It should be mentioned that the immunohistological demonstration of RVFV proteins was increased in multiple organs in *A.ae.* mosquitoes, notwithstanding that the mosquito specimens exhibited more or less a similar distribution pattern of RVFV. Hence, there must be a limiting factor of infection in *D.mel.* flies and *Cx.qu.* mosquitoes in comparison to *A.ae.* mosquitoes. Dietrich et al.^70^ demonstrated similar findings by investigating the abundance of viRNAs, which were markedly reduced in *Cx.qu.* in comparison to *A.ae.* Here, a lower infection status was discussed. However, this needs to be investigated on a molecular level in future studies.

A critical aspect of this study is the marked cross-reactivity between various antibodies and examined specimens, highlighting the importance of a critical analysis of immunoreactivity and application of adequate negative controls. This cross-reactivity of various virus specific antibodies within host tissue needs to be taken into consideration while performing research with RVFV or during routine diagnostics. The regular false positive labeling of cortical cells in the head ganglia of mosquitoes and fruit flies is particularly striking. Similar observations were described for the fat body and nephrocytes in RVFV-infected *Culex pipiens*^86^, which in part were also observed in the present study and should therefore be considered as unspecific. The same applies for a diffuse staining pattern of applied antibodies. Moreover, there was a strong immunoreactivity within the liver of mock-infected mammals using the antibodies Np9, Gc9A9, polyGc and polyGn. In ovine tissue, there was a severe cross reactivity between the above-named antibodies and antigen-presenting cells.

The results provide highly needed, comparable insights of RVFV distribution and antibody usefulness for immunohistological investigations. The antibodies used in the present study, in particular anti-nucleoprotein and -glycoprotein antibodies are suitable to detect RVFV in tissues of multiple species. While it is recommended to use nucleoprotein-targeted antibodies for general diagnostic detection of RVF, glycoprotein-targeted antibodies pose a competent alternative, especially in insect tissue. Nonetheless, different antigens may be targets within scientific investigations of RVF pathogenesis. Therefore, the antibodies directed against non-structural RVFV proteins represent a promising tool for future studies, especially in mosquitoes. However, when applying different antibodies in different species, the possibility of an unspecific or false positive immunostaining should be considered and accurate analysis requires inclusion of appropriate controls. Conclusively, the antibodies investigated within the study represent a valuable tool for further diagnostic and scientific use in RVFV detection and research.

## Materials and methods

### RVFV origin

RVFV strain MP12 used for C6/36 cells, insects, and mice (Richard Elliott and Benjamin Brennan, Institute of Infection, Immunity and Inflammation, Centre for Virus Research, University of Glasgow, Glasgow, UK) was propagated using Vero-E6-cells (Collection of Cell Lines in Veterinary Medicine (CCLV), #CCLV-RIE 929, Friedrich-Loeffler-Institute (FLI), Riems, Germany) on a 96-well plate in Dulbecco’s Modified Eagle’s Medium (DMEM, #DMEM-HXA, Capricorn Scientific GmbH, Ebsdorfergrund, Germany) / 5% fetal bovine serum (FBS; #FBS-HI-12A FBS, Capricorn Scientific GmbH, Ebsdorfergrund, Germany) at a temperature of 33 °C in a humidified atmosphere and a CO_2_-content of 5%. Supernatant of infected cells was harvested after 3 days with cells showing a cytopathic effect (CPE), characterized by cell lysis in 80% of cells. Additionally, mice and sheep were infected with the virulent RVFV 35/74 strain provided by the virus stock of the FLI, Riems, Germany. The strain was propagated in a cell culture flask of BHK21 cells using Minimum Essential Medium (MEM) supplemented with penicillin–streptomycin and 2% FBS (FLI Bio-Bank, FLI, Riems, Germany) at a temperature of 37 °C and 5% CO_2_. Supernatant was harvested, when cells showed 80% CPE. A TCID_50_ assay, calculated as described by Spearman and Kaerber^81^, was used to determine the virus titer.

### Tissue samples

All utilized specimens were part of different, RVF-related animal experiments, which were approved and authorized by the responsible animal welfare officers and the local authorities [Landesamt für Landwirtschaft, Lebensmittelsicherheit und Fischerei Mecklenburg-Vorpommern (permissions 7221.3-1-038/17 and 7221.3-1.1-048/17)] and performed in accordance with the German regulations and legal requirements.

C6/36 cells were obtained from the FLI (CCLV, #CCLV-RIE_1299, FLI, Riems, Germany) and maintained in T75 tissue culture flasks (#83.3911.002, Sarstedt, Germany) with Schneider's drosophila medium (#P04-90500, PAN Biotech, Aidenbach, Germany)/10% FBS (#S181H, Biowest, Riverside, USA)/1% penicillin–streptomycin/1% Gln/1% non-essential amino acids (#P08-32100, Biochrom, Berlin, Germany)/1% sodium pyruvate (#P04-43100, Pan Biotech, Aidenbach, Germany) in an incubator at a temperature of 25 °C. For further analyses, cells were scraped, washed in phosphate buffer saline (PBS) and centrifuged at 300×*g* for 5 min to form cell pellets of uniform size and cellular density^78^.

*A.ae.* (Bayer, Leverkusen, Germany) and *Cx.qu.* mosquitoes (origin Malaysia, courtesy of Bayer, Leverkusen, Germany), reared at the Bernhard Nocht Institute for Tropical Medicine (BNITM) in Hamburg, Germany, served as infection models. Specimens were kept in insectaries with a 12 h:12 h light:dark photoperiod, a relative humidity of 80% and at a temperature of 26 °C. Emerged mosquitoes received fructose pads (8% D-Fructose, #4981.4, Carl Roth GmbH, Karlsruhe, Germany; 0.02% 4-Aminobenzoic acid, #A9878-5G, Sigma Aldrich, Seelze, Germany) *ad libitum*. Females were additionally fed with a blood meal containing concentrated human erythrocytes (Blood group 0, Blood bank, University Hospital Hamburg, expired)/50% fetal bovine serum (FBS; FBS-Standard, Pan Biotech, Aidenbach, Germany) for egg production. Egg rafts of the species *Culex* were kept in tap water. Egg rafts of the genus *Aedes* were dried for 14 days and afterwards placed in tap water. Hatched larvae were reared with fish food tablets (Astra fish food, Astra Aquaria GmbH, Hameln, Gemany).

Laboratory strains of *D.mel. cnbw and yw* (Jean-Luc Imler, Institut de Biologie Moléculaire et Cellulaire; Université Luis Pasteur, Strasbourg) were bred in the Research Center for Emerging Infections and Zoonoses (RIZ, University for Veterinary Medicine, Hannover). Fruit flies were kept in an environment without light at 25 °C and 65% relative humidity and received *Drosophila* food (#66-116, NutriFly-Bloomington formulation; Genesee Scientific, El Cajon, CA, USA). Prior to further sample preparation, mosquitoes and fruit flies were anesthetized with carbon dioxide for further tissue preparation.

Sheep were obtained from FLI sheep flock in Mariensee (Germany), checked for their health status and kept at the FLI, Riems, Germany under BSL 3 conditions. They received water *ad libitum* and were fed with hay pellets and concentrate (CeravisAG, Rendsburg, Germany). For euthanasia, animals were at first sedated with a combination of xylazine (CP-Pharma Handelsgesellschaft mbH, Burgdorf, Germany) and ketamine (Serumwerk Bernburg AG, Bernburg, Germany) and finally euthanized with embutramide (T61, MSD, Kenilworth, New Jersey, USA) according to manufacturer’s instructions. Thereafter, liver samples were obtained during necropsy.

C57Bl/6-IFNAR^tmAgt^ mice were bred in and obtained from the FLI mouse stock, while heterozygous Crl:NU(NCr)-*Foxn1*^*nu*^ mice were commercially obtained from Charles River Laboratories. Both strains, kept at the FLI in isolated ventilated cages, received water *ad libitum* and were fed with standard mouse food (ssniff Spezialdiäten GmbH, Soest, Germany). Prior necropsy for obtaining liver specimen, mice were euthanized at given criteria by isoflurane anesthesia and subsequent cardiac blood drain.

### Animal and C6/36 cell infection

1.6 × 10^7^ C6/36 cells were infected with a TCID_50_ of 2.3 × 10^6^/ml of RVFV strain MP12 diluted in Schneider's drosophila medium and incubated for 24 h at 25 °C. 5–7 day old *A.ae.* and *Cx.qu.* as well as 5–7 day old *D.mel.* (strain *cnbw* and *yw*) were infected by a lateral injection into the thorax via glass capillary (Nanoject II Drummond, Drummond Scientific Company, Broomall, PA). While mosquitoes received 27.6 nl with a TCID_50_ of 1.6 × 10^3^/ml/specimen, fruit flies were inoculated with 23 nl with a TCID_50_ of 3.3 × 10^3^/ml/specimen of RVFV strain MP12. Mosquito as well as fruit fly mock-infected controls received 27.6 nl and 9.2 nl Schneider’s drosophila medium from BHK21 cells, respectively. Thereafter, mosquitoes and fruit flies were kept for another 5 days to guaranty establishment of RVFV infection. While mosquitoes were maintained at 27 °C ± 5 °C with a relative humidity of 70%, fruitflies were kept at 25 °C with a relative humidity of 65%.

Adult sheep received an intramuscular injection of 1 ml virus suspension with a TCID_50_ of 10^5^/ml of RVFV strain 35/74. C57Bl/6-IFNAR^tmAgt^ mice were infected by a subcutaneous injection of 0.1 ml virus suspension with a TCID_50_ of 1.4 × 10^3^/animal of RVFV MP12, while heterozygous Crl:NU(NCr)-*Foxn1*^*nu*^ mice received the same amount of RVFV 35/74. Likewise, negative controls were mock-infected by subcutaneous application of virus-free MEM. After infection, sheep were observed for four days and then euthanized to analyze virus distribution in an early stage of RVFV infection. Mice were euthanized after developing symptoms of severe disease according to an animal welfare score, which resulted in 2–4 days post infection.

### Control of infection status

For the verification of the infection status, fruit fly brains (n = 5) were homogenized in 500 µl Schneider's drosophila medium and viral RNA was purified with QIAamp Viral RNA Mini Kit (#52904, Qiagen, Hilden, Germany) according to the manufacturer’s instructions. Additionally, one individual per mosquito species (n = 1) and four individuals per fruit fly species (n = 4) of RVFV-infected and mock-infected FFPE specimens were homogenized, deparaffinized in xylol (#9713.2, Carl Roth GmbH and Co. KG, Karlsruhe, Germany) and subsequently purified using RNeasy FFPE Kit (#73504, Qiagen, Hilden, Germany) according to the manufacturer’s instructions. RT-qPCR was performed using Qiagen One Step RT-PCR Kit (#210212, Qiagen, Hilden, Germany) and the AriaMX real-time PCR system (Agilent Technologies Deutschland GmbH, Waldbronn) using 0.6 μM of the following primers and 0.2 µM of probe: RVFV-F (OSM_162, sense, TGA AAA TTC CTG AAA CAC ATG G), RVFL-R (OSM_93, antisense, ACT TCC TTG CAT CAT CTG ATG) and RFVL-probe (OSM_94, CAA TGT AAG GGG CCT GTG TGG ACT TGT G) as previously published^70^. Synthetic RNA comprising the target region of the RT-qPCR was used as positive control, while a water sample was used as negative control.

For evaluation of mice and sheep by RT-qPCR, liver tissue was lysed in cell culture medium using the Qiagen TissueLyser II (Qiagen, Hilden, Germany). After centrifugation, RNA was isolated from supernatant with the NucleoMag VET Kit (Machery & Nagel, Düren, Germany) in the automated KingFisher Flex Purification System (Thermo Scientific, Waltham, USA). Samples were tested once in RT-qPCR according to a previously published RVFV protocol^87^.

### Histopathology and immunohistochemistry

Four individuals per insect species, (two in sagittal and transversal plane each; n = 2 + 2), and two murine and ovine liver samples each (n = 2) of infected and mock-infected specimens, respectively, served for histological assessment. One C6/36 cell pellet (n = 1) functioned as a system positive control in this study. Insect specimens were fixed in 10% neutral buffered formalin for 24 h, while mammal tissue samples were fixed in formalin for 21 days. Thereafter, samples were embedded in paraffin wax and routinely cut to generate 2–4 µm thick sections used for routine hematoxylin–eosin (HE) staining and immunolabeling.

Evaluation of histopathology was performed on HE-stained sections^88^, generated by means of an automated slide stainer (Leica ST 4040; Leica Biosystems, Germany).

Regarding immunohistochemistry, a panel of various mono- and polyclonal antibodies against different Rift Valley fever virus antigens including viral nucleoproteins, glycoproteins and non-structural proteins (detailed in Table 1) were evaluated. All antibodies were initially tested using different concentrations and pretreatments including proteinase K. The concentration and pretreatment with the highest efficacy were further employed as previously described^89^.

Samples were demasked in simmering citrate buffer (pH: 6; #3958.1, Carl Roth GmbH & Co. KG, Karlsruhe, Germany) for 20 min using a microwave (Privileg 8020, 800 W) or received no pretreatment. In order to prevent non-specific binding, samples were incubated with either goat or rabbit serum, diluted 1:5 in phosphate buffered saline (pH 7.2). Thereafter, samples were incubated overnight at 4 °C with primary antibodies, diluted in PBS with bovine serum albumin (Albumin Fraktion V, #0163.2, Carl Roth GmbH & Co. KG, Karlsruhe, Germany). Biotinylated goat-anti-mouse (1:200; #BA-9200, VECTOR, Biozol Diagnostica Vetrieb GmbH, Eching, Germany), goat-anti-rabbit (1:200; #BA-1000, VECTOR, Biozol Diagnostica Vetrieb GmbH, Eching, Germany) and rabbit-anti-sheep (1:200; #BA-600, VECTOR, Biozol Diagnostica Vetrieb GmbH, Eching, Germany) antibodies served as secondary antibodies, respectively. Visualization was achieved by the use of the avidin–biotin–peroxidase complex (#PK 6100, Vectastain elite ABC kit, Vector Laboratories, Burlingame, USA) with 3,3′-diaminobenzidine tetrahydrochloride (DAB, #32750 25GF, Sigma Aldrich Chemie GmbH, Tauffkirchen, Germany) according to the manufacturer’s protocol. Final section preparation included counterstaining with Mayer’s hematoxylin (#T865.2, Carl Roth GmbH & Co. KG, Karlsruhe, Germany) and mounting with RotiHistokittII (#T160.1, Carl Roth GmbH and Co. KG, Karlsruhe, Germany).

Primary antibodies were either replaced with ascites fluid from non-immunized BALB/c mice (1:1000; #BL CL8100, Cedarlane, biologo, Kronshagen, Germany), sheep normal serum (1:3000, serum of sheep from the Clinic for Swine, Small Ruminants and Forensic Medicine, University of Veterinary Medicine Hannover, Germany) or rabbit normal serum (1:3000; #R4505, Sigma-Aldrich Chemie GmbH, Tauffkirchen, Germany) in antibody negative controls. Furthermore, cross-reactivity of primary and secondary antibodies along with the ABC Vectorstain kit and DAB was examined in performing the immunohistological experiment as described above but omitting one reagent each in separate experimental runs.

### Evaluation of results

Tissue sections were evaluated regarding microscopic lesions and immunolabeling by three pathologists using light microscopy (OLYMPUS BX53; Olympus Europa SE & Co. KG, Hamburg, Germany). Immunoreactivity of positive cell immunolabeling was semiquantitatively estimated in mice, sheep and the C6/36 cell pellet: mild for < 30% (+), moderate for 30–60% (++) and marked for > 60% (+++) labeled cells per high power field. The distribution and morphology of immunolabeling in insects in vivo, was classified as either positive (+) or negative (−) with respect to different organ involvement.

## Supplementary Information

Supplementary Information 1.

Supplementary Information 2.

Supplementary Information 3.

Supplementary Information 4.

Supplementary Information 5.

Supplementary Information 6.

Supplementary Information 7.

Supplementary Information 8.

Supplementary Information 9.
