# Supplementary material for: Rift Valley fever virus detection in susceptible hosts with special emphasis in insects
Source: Sci Rep. 2021 May 10;11:9822. doi: 10.1038/s41598-021-89226-z (PMC8110843; doi:10.1038/s41598-021-89226-z)
Supplement: Supplementary file 1 — Supplementary Information 1. [file 41598_2021_89226_MOESM1_ESM.docx]

# **Rift Valley fever virus detection in susceptible hosts with special emphasis in insects**

K.M. Gregor^1,7^, L.M. Michaely^1,7^, B. Gutjahr^2,7^, M. Rissmann^2^, M. Keller^2^, S. Dornbusch^3^, F. Naccache^3^, K. Schön^3^, S. Jansen^4^, A. Heitmann^4^, R. König^2^, B. Brennan^5^, R.M. Elliott^5^, S. Becker^3^, M. Eiden^2^, I. Spitzbarth^6^, W. Baumgärtner^1^, C. Puff^1,8^, R. Ulrich^2,6,8^, M.H. Groschup^2,8^

^1^ Department of Pathology, University of Veterinary Medicine Hannover;

^2^ Institute of Novel and Emerging Infectious Diseases, Friedrich-Loeffler-Institute, Greifswald;

^3^ Institute for Parasitology and Research Center for Emerging Infections and Zoonoses, University of Veterinary Medicine Hannover;

^4^ Department of Arbovirology, Bernhard Nocht Institute for Tropical Medicine, Hamburg, Germany

^5^ MRC-University of Glasgow Centre for Virus Research, Glasgow, Scotland, United Kingdom

^6^ Institute of Veterinary Pathology, Faculty of Veterinary Medicine, Leipzig University;

^7^ These authors contributed equally: K.M. Gregor, L.M. Michaely and B. Gutjahr

^8^ These authors jointly supervised this work: C. Puff, R. Ulrich and M.H. Groschup

* Corresponding author: Prof. Wolfgang Baumgärtner, Department of Pathology, University of Veterinary Medicine Hannover, E-mail address: Wolfgang.Baumgaertner@tiho-hannover.de

# Supplementary information

S1. Pathology

1. S2. Rift Valley fever nucleotide acid sequence of target epitopes
2. Supplementary Figures S1-S176
3. References

# S1. Pathology

In addition to the assessment of antibodies, samples were investigated with regard to histopathologic lesions. In RVFV infected as well as mock-infected *Cx.qu.* and *A.ae.,* trophocytes had enlarged nuclei with a distinct, medium-sized, round, eosinophilic nucleolus. One individual of *Cx.qu*. exhibited severe lesions with thoracic ganglia either lost or scattered and dislocated within the thorax (see Fig. 2, 8, 14). Normally, three thoracic ganglia pairs are located dorsally of the legs. Only remnants of cortical cells and neuropil of the two caudal pairs were observed at the anatomical locus, while the cranial pair was relocated with ventral lobes of the salivary gland to a craniodorsal position. Moreover, RVFV infected mosquito specimens as well as mock-infected *D.mel. cnmb* and *yw* presented microscopic lesions of varying degree, including degeneration (fragmentation), necrosis (karyorrhexis, cellular debris) and dislocation of flight musculature (see Supplementary Fig. S45, S68, S90). No histological changes were observed in the C6/36 cell pellet.

Histological lesions in liver specimens from mice were characterized by diffuse, hepatocellular necrosis. In contrast, an irregular, multifocal, perivascular, lympho-histiocytic hepatitis was present in HE-stained liver from sheep.

Infection and dissemination assays along with consecutive pathological changes following RVFV inoculation already underwent intense research in the past decades. The here observed changes in murine and ovine tissue were reminiscent by previous studies^1-7^. Interestingly, to date no pathohistological changes have been described in *Culex sp.*^8-10^ or *Aedes sp*.^11,12^. Regarding insect specimen, reactive changes were observed within the present study in the form of enlarged nuclei within trophocytes in both mosquito species. Thus, these results might support previous observations of decreased fertility, efficacy to feed^13^ or survive after infection^14,15^. Romoser et al. (1992) argumented, that these observations could be related to the infection of 1) the fat body as an important organ for vitellogenesis and survival and 2) the infection of the nervous system, including neurosecretory cells, as the main regulatory organ system^9^. Therefore, the observed lesions could represent a morphological correlate to RVFV infection. Since mock-infected individuals presented the same changes, it cannot be excluded that they represent typical changes caused by the mode of inoculation. The observed pathological changes in the flight musculature in examined RVFV-infected mosquitoes and mock-infected fruit flies as well as the thorax ganglia in one individual of *Cx.qu.* are considered to be a consequence of the intrathoracic injection. Therefore, these lesions are not initially associated with the RVFV infection in this study. Noteworthy, the described observations do not reflect natural RVFV pathogenesis given the fact that this RVFV infection assay investigated intrathoracically infected insect specimens.

# S2. Rift Valley fever nucleotide acid sequence of target epitopes

Table S1: Known target epitope data.

| **Target epitope** | **Nucleotide sequence** | **Nucleotide position** |
| --- | --- | --- |
| Gn164b | EDPHLRNRPGKGHNYIDGMT | #DQ380208: 480-539 |
| Gc9a9 | ERPLLVKGTLIAIDPFDDRR | #DQ380208: 3333-3392 |
| 7B6^16^ | KGTMDSGQTKR | #DQ380208: 1164-1196 |

# Figure S1-S22: Comparison of epitope expression in C6/36 cell pellet

**Figure S1-22:** Comparison of epitope expression in MP12-infected C6/36 cell pellet with intracytoplasmic (arrowheads) or intranuclear (arrows), granular signals for Rift Valley fever virus (RVFV). S1-2: Hematoxylin-Eosin (HE) stain of infected (S1) and non-infected (S2) C6/36 cells. S3-4: Np9 immunohistology on infected (S3) and non-infected (S4) C6/36 cells. S5-6: S24Np immunohistology on infected (S5) and non-infected (S6) C6/36 cells. S7-8: polyNp immunohistology on infected (S7) and non-infected (S8) C6/36 cells. S9-10: Gc9A9 immunohistology on infected (S9) and non-infected (S10) C6/36 cells. S11-12: polyGc immunohistology on infected (S11) and non-infected (S12) C6/36 cells. S13-14: Gn164b immunohistology on infected (S13) and non-infected (S14) C6/36 cells. S15-16: 7B6 immunohistology on infected (S15) and non-infected (S16) C6/36 cells. S17-18: polyGn immunohistology on infected (S17) and non-infected (S18) C6/36 cells. S19-20: NSs5F12 immunohistology on infected (S19a, b) and non-infected (S20) C6/36 cells. S21-22: NSm1E9A2 immunohistology on infected (S21) and non-infected (S22) C6/36 cells.

# Figure S23-S44: Comparison of epitope expression in Culex quinquefasciatus

**Figure S23-44:** Comparison of epitope expression in MP12-infected *Culex quinquefasciatus* (*Cx.qu.*) with intracytoplasmic (arrowheads) or intranuclear (arrows), granular signals for RVFV. S23-24: HE stain of infected (S23) and mock-infected (S24) *Cx.qu.* S25-26: Np9 immunohistology on infected (S25a, b) and mock-infected (S26) *Cx.qu.* S27-28: S24Np immunohistology on infected (S27a, b) and mock-infected (S28) *Cx.qu.* S29-30: polyNp immunohistology on infected (S29a, b) and mock-infected (S30) *Cx.qu.* S31-32: Gc9A9 immunohistology on infected (S31a, b) and mock-infected (S32) *Cx.qu.* S33-34: polyGc immunohistology on infected (S33a, b) and mock-infected (S34) *Cx.qu.* S35-36: Gn164b immunohistology on infected (S35a, b) and mock-infected (S36) *Cx.qu.* S37-38: 7B6 immunohistology on infected (S37a, b) and mock-infected (S38) *Cx.qu.* S39-40: polyGn immunohistology on infected (S39a, b) and mock-infected (S40) *Cx.qu.* S41-42: NSs5F12 immunohistology on infected (S41a, b) and mock-infected (S42) *Cx.qu.* S43-44: NSm1E9A2 immunohistology on infected (S43a, b) and mock-infected (S44) *Cx.qu.* a: aorta, ag: abdominal ganglia, am: anterior midgut, c: cardia, d: diverticulum, e: esophagus, fm: flight muscle, h: hindgut, he: heart, hg: head ganglia, l: legs, mt: Malpighian tubules, om: ommatidia, ov: ovary, pm: posterior midgut, sg: salivary gland, tg: thoracic ganglia, t: trophocytes.

# Figure S45-S66: Comparison of epitope expression in Aedes aegypti

**Figure S45-66:** Comparison of epitope expression in MP12-infected *Aedes aegypti* (*A.ae.*) with intracytoplasmic (arrowheads) or intranuclear (arrows), granular signals for RVFV. S45-46: HE stain of infected (S45) and mock-infected (S46) *A.ae.* Note the fragmented and necrotic flight musculature (S45, asterisk). S47-48: Np9 immunohistology on infected (S47) and mock-infected (S48) *A.ae.* S49-50: S24Np immunohistology on infected (S49) and mock-infected (S50) *A.ae.* S51-52: polyNp immunohistology on infected (S51) and mock-infected (S52) *A.ae.* S53-54: Gc9A9 immunohistology on infected (S53) and mock-infected (S54) *A.ae.* S55-56: polyGc immunohistology on infected (S55) and mock-infected (S56) *A.ae.* S57-58: Gn164b immunohistology on infected (S57) and mock-infected (S58) *A.ae.* S59-60: 7B6 immunohistology on infected (S59) and mock-infected (S60) *A.ae.* S61-62: polyGn immunohistology on infected (S61) and mock-infected (S62) *A.ae.* S63-64: NSs5F12 immunohistology on infected (S63) and mock-infected (S64) *A.ae.* S65-66: NSm1E9A2 immunohistology on infected (S65) and mock-infected (S66) *A.ae.* a: aorta, ag: abdominal ganglia, am: anterior midgut, c: cardia, d: diverticulum, e: esophagus, fm: flight muscle, g: gonoduct, h: hindgut, he: heart, hg: head ganglia, jo: Johnston’s organ, l: legs, mt: Malpighian tubules, o: oenocytes, om: ommatidia, ov: ovary, p: pharynx, pm: posterior midgut, r: rectal papillae, s: seminal vesicle, sg: salivary gland, tg: thoracic ganglia, t: trophocytes.

# Figure S67-S88: Comparison of epitope expression in Drosophila melanogaster cinnabar-brown

**Figure S67-88:** Comparison of epitope expression in MP12-infected *Drosophila melanogaster cinnabar-brown* (*D.mel. cnbw*) with intracytoplasmic (arrowheads), granular signals for RVFV. S67-68: HE stain of infected (S67) and mock-infected (S68) *D.mel. cnbw*. Note the fragmented, necrotic and dislocated flight musculature (S68, asterisk). S69-70: Np9 immunohistology on infected (S69) and mock-infected (S70) *D.mel. cnbw*. S71-72: S24Np immunohistology on infected (S71) and mock-infected (S72) *D.mel. cnbw*. S73-74: polyNp immunohistology on infected (S73) and mock-infected (S74) *D.mel. cnbw*. S75-76: Gc9A9 immunohistology on infected (S75) and mock-infected (S76) *D.mel. cnbw*. S77-78: polyGc immunohistology on infected (S77) and mock-infected (S78) *D.mel. cnbw*. S79-80: Gn164b immunohistology on infected (S79) and mock-infected (S80) *D.mel. cnbw*. S81-82: 7B6 immunohistology on infected (S81) and mock-infected (S82) *D.mel. cnbw*. S83-84: polyGn immunohistology on infected (S83) and mock-infected (S84) *D.mel. cnbw*. S85-86: NSs5F12 immunohistology on infected (S85) and mock-infected (S86) *D.mel. cnbw.* S87-88: NSm1E9A2 immunohistology on infected (S87) and mock-infected (S88) *D.mel. cnbw.* a: aorta, am: anterior midgut, c: cardia, d: diverticulum, e: esophagus, fm: flight muscle, g: gonoduct, h: hindgut, hg: head ganglia, jo: Johnston’s organ, l: legs, mt: Malpighian tubules, o: oenocytes, om: ommatidia, ov: ovary, pm: posterior midgut, r: rectal papillae, s: seminal vesicle, tg: thoracic ganglia, t: trophocytes.

# Figure S89-S110: Comparison of epitope expression in Drosophila melanogaster yellow-white

**Figure S89-110:** Comparison of epitope expression in MP12-infected *Drosophila melanogaster yellow-white* (*D.mel. yw*) with intracytoplasmic (arrowheads), granular signals for RVFV. S89-90: HE stain of infected (S89) and mock-infected (S90) *D.mel. yw.* Note the fragmented, necrotic and dislocated flight musculature (S90, asterisk). S91-92: Np9 immunohistology on infected (S91) and mock-infected (S92) *D.mel. yw.* S93-94: S24Np immunohistology on infected (S93) and mock-infected (S94) *D.mel. yw.* S95-96: polyNp immunohistology on infected (S95) and mock-infected (S96) *D.mel. yw*. S97-98: Gc9A9 immunohistology on infected (S97) and mock-infected (S98) *D.mel. yw*. S99-100: polyGc immunohistology on infected (S99) and mock-infected (S100) *D.mel. yw.* S101-102: Gn164b immunohistology on infected (S101) and mock-infected (S102) *D.mel. yw*. S103-104: 7B6 immunohistology on infected (S103) and mock-infected (S104) *D.mel. yw*. S105-106: polyGn immunohistology on infected (S105) and mock-infected (S106) *D.mel. yw*. S107-108: NSs5F12 immunohistology on infected (S107) and mock-infected (S108) *D.mel. yw*. S109-110: NSm1E9A2 immunohistology on infected (S109) and mock-infected (S110) *D.mel. yw*. a: aorta, am: anterior midgut, c: cardia, d: diverticulum, e: esophagus, fm: flight muscle, g: gonoduct, h: hindgut, hg: head ganglia, jo: Johnston’s organ, l: legs, mt: Malpighian tubules, o: oenocytes, om: ommatidia, ov: ovary, pm: posterior midgut, r: rectal papillae, s: seminal vesicle, tg: thoracic ganglia, t: trophocytes.

# Figure S111-S132: Comparison of epitope expression in sheep

**Figure S111-132:** Comparison of epitope expression in 35/74-infected sheep with intracytoplasmic, granular signals for RVFV. S111-112: HE stain of infected (S111) and mock-infected (S112) sheep. Note the multifocal, circumscribed lympho-histiocytic infiltrates within lesions (S111). S113-114: Np9 immunohistology on infected (S113) and mock-infected (S114) sheep. S115-116: S24Np immunohistology on infected (S115) and mock-infected (S116) sheep. S117-118: polyNp immunohistology on infected (S117) and mock-infected (S118) sheep. S119-120: Gc9A9 immunohistology on infected (S119) and mock-infected (S120) sheep. S121-122: polyGc immunohistology on infected (S121) and mock-infected (S122) sheep. S123-124: Gn164b immunohistology on infected (S123) and mock-infected (S124) sheep. S125-126: 7B6 immunohistology on infected (S125) and mock-infected (S126) sheep. S127-128: polyGn immunohistology on infected (S127) and mock-infected (S128) sheep. S129-130: NSs5F12 immunohistology on infected (S129) and mock-infected (S130) sheep. S131-132: NSm1E9A2 immunohistology on infected (S131) and mock-infected (S132) sheep.

# Figure S133-S154: Comparison of epitope expression in CrlNU(NCr)-Foxn-1^nu^ mice

**Figure S133-154:** Comparison of epitope expression in 35/74-infected CrlNU(NCr)-Foxn-1^nu^ mice with intracytoplasmic, granular signals for RVFV. S133-134: HE stain of infected (S133) and mock-infected (S134) CrlNU(NCr)-Foxn-1^nu^ mice, S135-136: Np9 immunohistology on infected (S135) and mock-infected (S136) CrlNU(NCr)-Foxn-1^nu^ mice, S137-138: S24Np immunohistology on infected (S137) and mock-infected (S138) CrlNU(NCr)-Foxn-1^nu^ mice, S139-140: polyNp immunohistology on infected (S139) and mock-infected (S140) CrlNU(NCr)-Foxn-1^nu^ mice, S141-142: Gc9A9 immunohistology on infected (S141) and mock-infected (S142) CrlNU(NCr)-Foxn-1^nu^ mice, S143-144: polyGc immunohistology on infected (S143) and mock-infected (S144) CrlNU(NCr)-Foxn-1^nu^ mice, S145-146: Gn164b immunohistology on infected (S145) and mock-infected (S146) CrlNU(NCr)-Foxn-1^nu^ mice, S147-148: 7B6 immunohistology on infected (S147) and mock-infected (S148) CrlNU(NCr)-Foxn-1^nu^ mice, S149-150: polyGn immunohistology on infected (S149) and mock-infected (S150) CrlNU(NCr)-Foxn-1^nu^ mice, S151-152: NSs5F12 immunohistology on infected (S151) and mock-infected (S152) CrlNU(NCr)-Foxn-1^nu^ mice, S153-154: NSm1E9A2 immunohistology on infected (S153) and mock-infected (S154) CrlNU(NCr)-Foxn-1^nu^ mice

# Figure S155-S176: Comparison of epitope expression in C57Bl6-IFNAR^tmAgt^ mice

**Figure S155-176:** Comparison of epitope expression in MP12-infected C57Bl6-IFNAR^tmAgt^ mice with intracytoplasmic, granular signals for RVFV. S155-156: HE stain of infected (S155) and mock-infected (S156) C57Bl6-IFNAR^tmAgt^ mice. Note the diffuse expression of Rift Valley fever antigen within necrotic hepatocytes (S155). S157-158: Np9 immunohistology on infected (S157) and mock-infected (S158) C57Bl6-IFNAR^tmAgt^ mice, S159-160: S24Np immunohistology on infected (S159) and mock-infected (S160) C57Bl6-IFNAR^tmAgt^ mice, S161-162: polyNp immunohistology on infected (S161) and mock-infected (S162) C57Bl6-IFNAR^tmAgt^ mice, S163-164: Gc9A9 immunohistology on infected (S163) and mock-infected (S164) C57Bl6-IFNAR^tmAgt^ mice, S165-166: polyGc immunohistology on infected (S165) and mock-infected (S166) C57Bl6-IFNAR^tmAgt^ mice, S167-168: Gn164b immunohistology on infected (S167) and mock-infected (S168) C57Bl6-IFNAR^tmAgt^ mice, S169-170: 7B6 immunohistology on infected (S169) and mock-infected (S170) C57Bl6-IFNAR^tmAgt^ mice, S171-172: polyGn immunohistology on infected (S171) and mock-infected (S172) C57Bl6-IFNAR^tmAgt^ mice, S173-174: NSs5F12 immunohistology on infected (S173) and mock-infected (S174) C57Bl6-IFNAR^tmAgt^ mice, S175-176: NSm1E9A2 immunohistology on infected (S175) and mock-infected (S176) C57Bl6-IFNAR^tmAgt^ mice.

# References

1 Smith, D. R. *et al.* The pathogenesis of Rift Valley fever virus in the mouse model. *Virol.* **407**, 256-267, doi:10.1016/j.virol.2010.08.016 (2010).

2 Ross, T. M., Bhardwaj, N., Bissel, S. J., Hartman, A. L. & Smith, D. R. Animal models of Rift Valley fever virus infection. *Virus Res.* **163**, 417-423, doi:10.1016/j.virusres.2011.10.023 (2012).

3 Ikegami, T. & Makino, S. The pathogenesis of Rift Valley fever. *Viruses* **3**, 493-519, doi:10.3390/v3050493 (2011).

4 Gerdes, G. H. Rift Valley fever. *Rev. Sci. Tech.* **23**, 613-623, doi: 10.20506/rst.23.2.1500 (2004).

5 Odendaal, L., Clift, S. J., Fosgate, G. T. & Davis, A. S. Lesions and cellular tropism of natural Rift Valley fever virus infection in adult sheep. *Vet. Pathol.* **56**, 61-77, doi:10.1177/0300985818806049 (2019).

6 Odendaal, L., Davis, A. S., Fosgate, G. T. & Clift, S. J. Lesions and cellular tropism of natural Rift Valley fever virus infection in young lambs. *Vet. Pathol.* **57**, 66-81, doi:10.1177/0300985819882633 (2020).

7 Daubney, R. & Hudson, J. R. Enzootic hepatitis or Rift Valley fever. *J. Pathol.* **34**, 545-579 (1931).

8 Faran, M. E., Romoser, W. S., Routier, R. G. & Bailey, C. L. The distribution of Rift Valley fever virus in the mosquito *Culex pipiens* as revealed by viral titration of dissected organs and tissues. *Am. J. Trop. Med. Hyg.* **39**, 206-213, doi: 10.4269/ajtmh.1988.39.206 (1988).

9 Romoser, W. S., Faran, M. E., Bailey, C. L. & Lerdthusnee, K. An immunocytochemical study of the distribution of Rift Valley fever virus in the mosquito *Culex pipiens*. *Am. J. Trop. Med. Hyg.* **46**, 489-501, doi: 10.4269/ajtmh.1992.46.489 (1992).

10 Romoser, W. S. *et al.* Pathogenesis of Rift Valley fever virus in mosquitoes--tracheal conduits & the basal lamina as an extra-cellular barrier. *Arch. Virol. Suppl.,* 89-100, doi:10.1007/3-211-29981-5_8 (2005).

11 Romoser, W. S. Studies of infection and dissemination of Rift Valley fever virus in mosquitoes: Annual report. Ohio Univ. Athens. https://apps.dtic.mil/sti/citations/ADA254744 (1989).

12 Romoser, W. S. *et al.* Rift Valley fever virus-infected mosquito ova and associated pathology: Possible implications for endemic maintenance. *Res. Rep. Trop. Med.* **2**, 121-127, doi:10.2147/RRTM.S13947 (2011).

13 Turell, M. J., Gargan, T. P. & Bailey, C. L. Replication and dissemination of Rift Valley fever virus in *Culex pipiens*. *Am. J. Trop. Med. Hyg.* **33**, 176-181, doi: 10.4269/ajtmh.1984.33.176 (1984).

14 Faran, M. E. *et al.* Reduced survival of adult *Culex pipiens* infected with Rift Valley fever virus. *Am. J. Trop. Med. Hyg.* **37**, 403-409, doi: 10.4269/ajtmh.1987.37.403 (1987).

15 Dohm, D. J., Romoser, W. S., Turell, M. J. & Linthicum, K. J. Impact of stressful conditions on the survival of *Culex pipiens* exposed to Rift Valley fever virus. *J. Am. Mosq. Control Assoc.* **7**, 621-623 (1991).

16 Keegan, K. & Collett, M. S. Use of bacterial expression cloning to define the amino acid sequences of antigenic determinants on the G2 glycoprotein of Rift Valley fever virus. *J. Virol.* **58**, 263-270, doi: 10.1128/JVI.58.2.263-270.1986 (1986).
