# Supplementary figures and images for: Rift Valley fever virus detection in susceptible hosts with special emphasis in insects
Source: Sci Rep. 2021 May 10;11:9822. doi: 10.1038/s41598-021-89226-z (PMC8110843; doi:10.1038/s41598-021-89226-z)

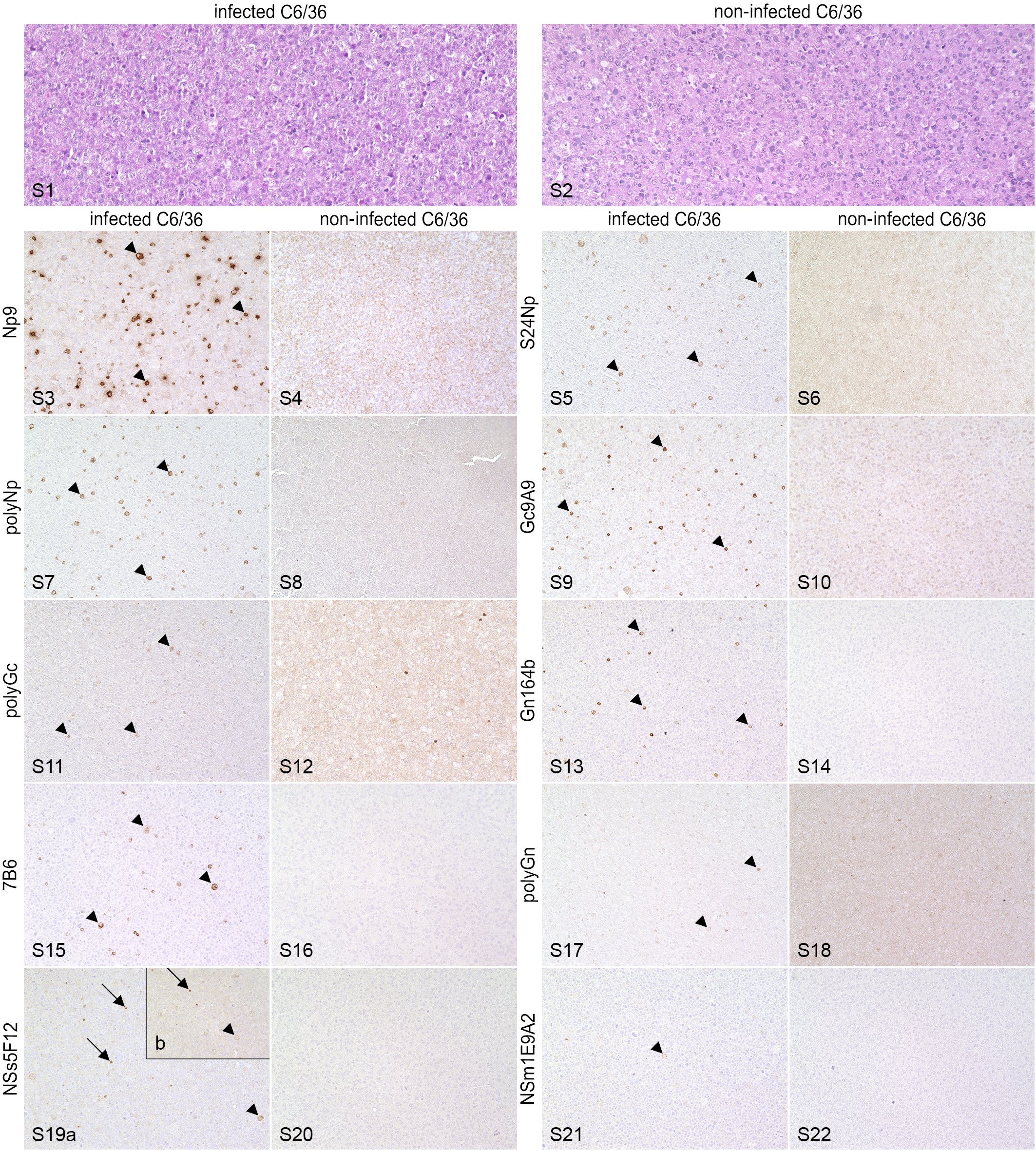

Supplement: Supplementary file 2 — Supplementary Information 2. [file 41598_2021_89226_MOESM2_ESM.tif]

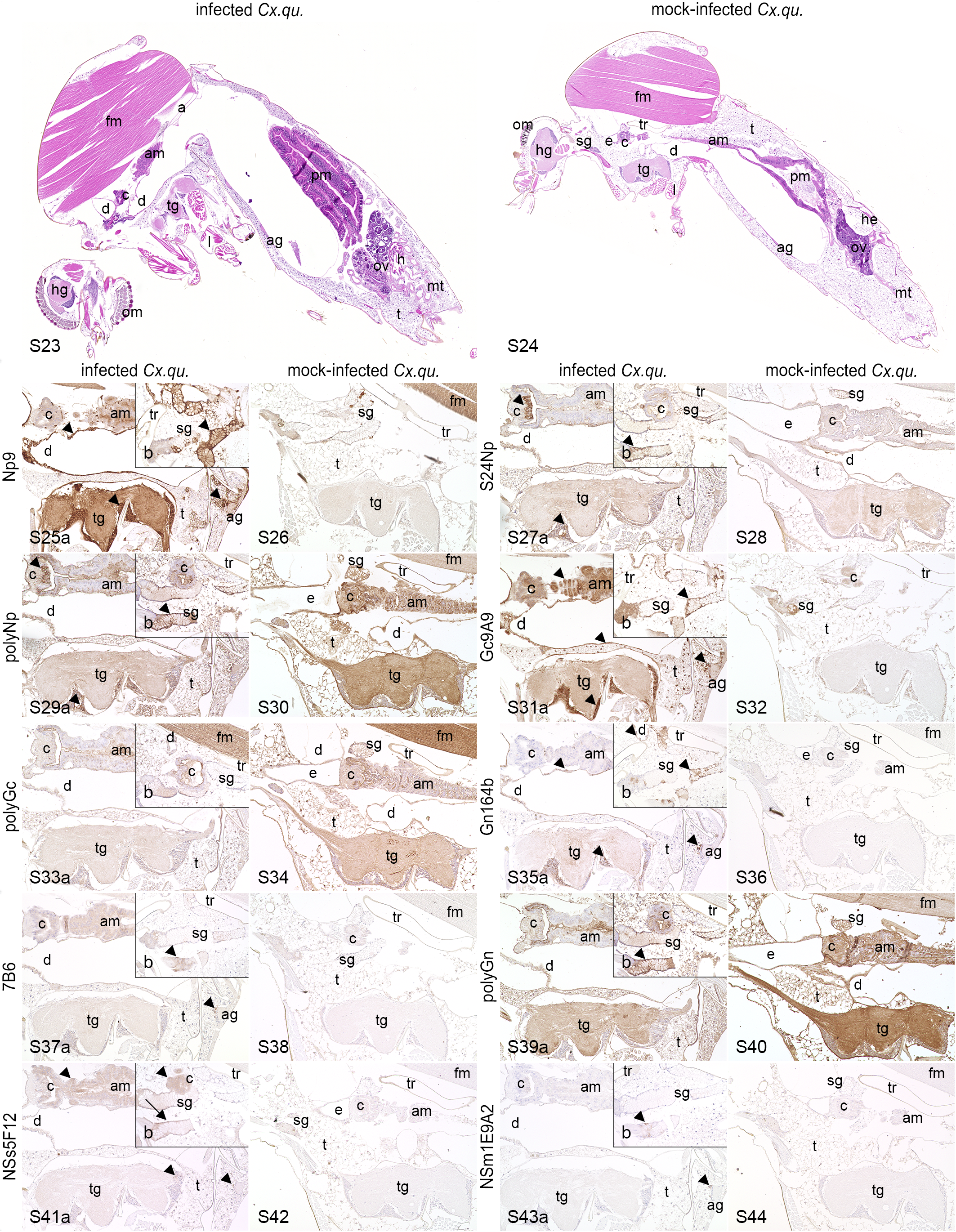

Supplement: Supplementary file 3 — Supplementary Information 3. [file 41598_2021_89226_MOESM3_ESM.tif]

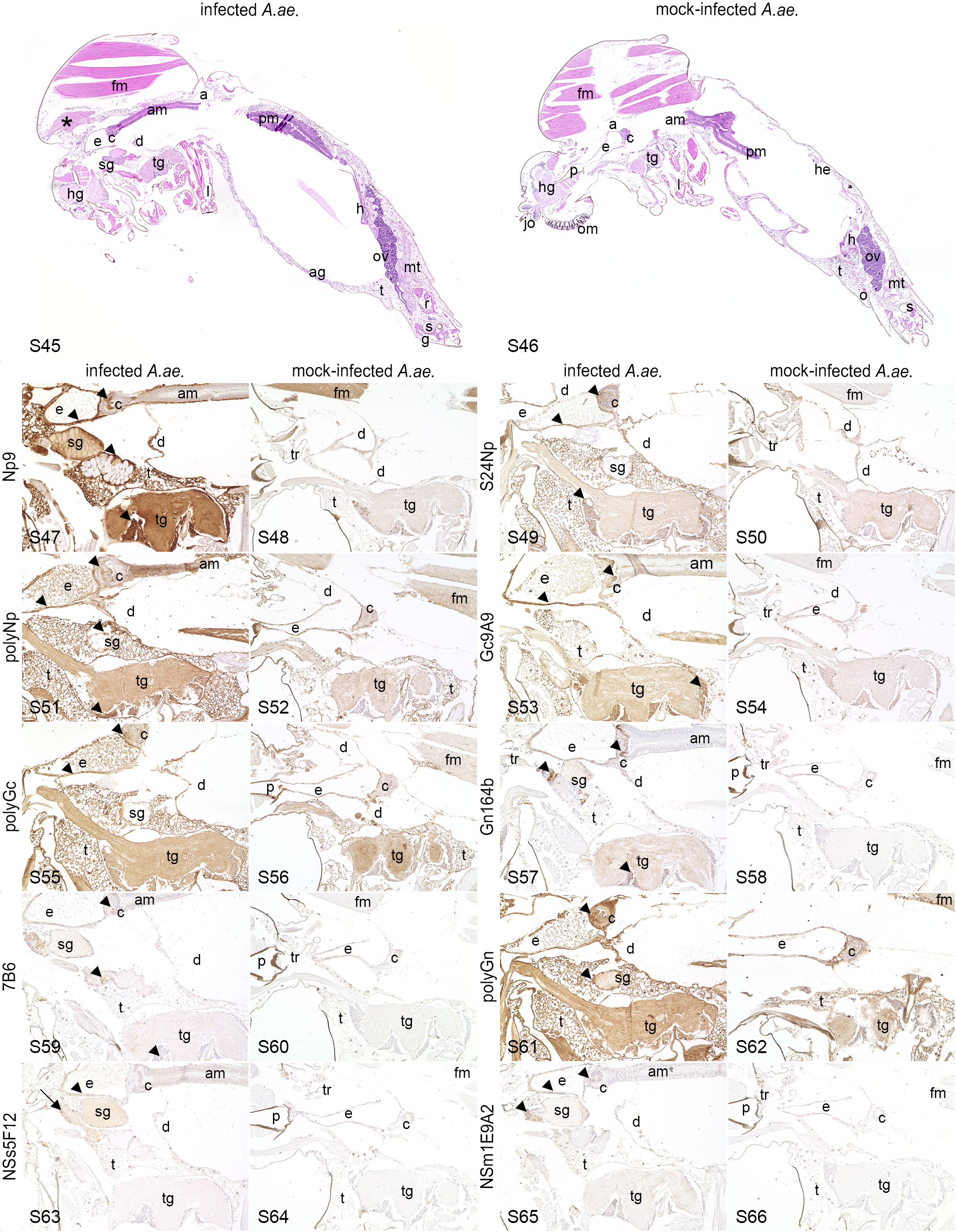

Supplement: Supplementary file 4 — Supplementary Information 4. [file 41598_2021_89226_MOESM4_ESM.tif]

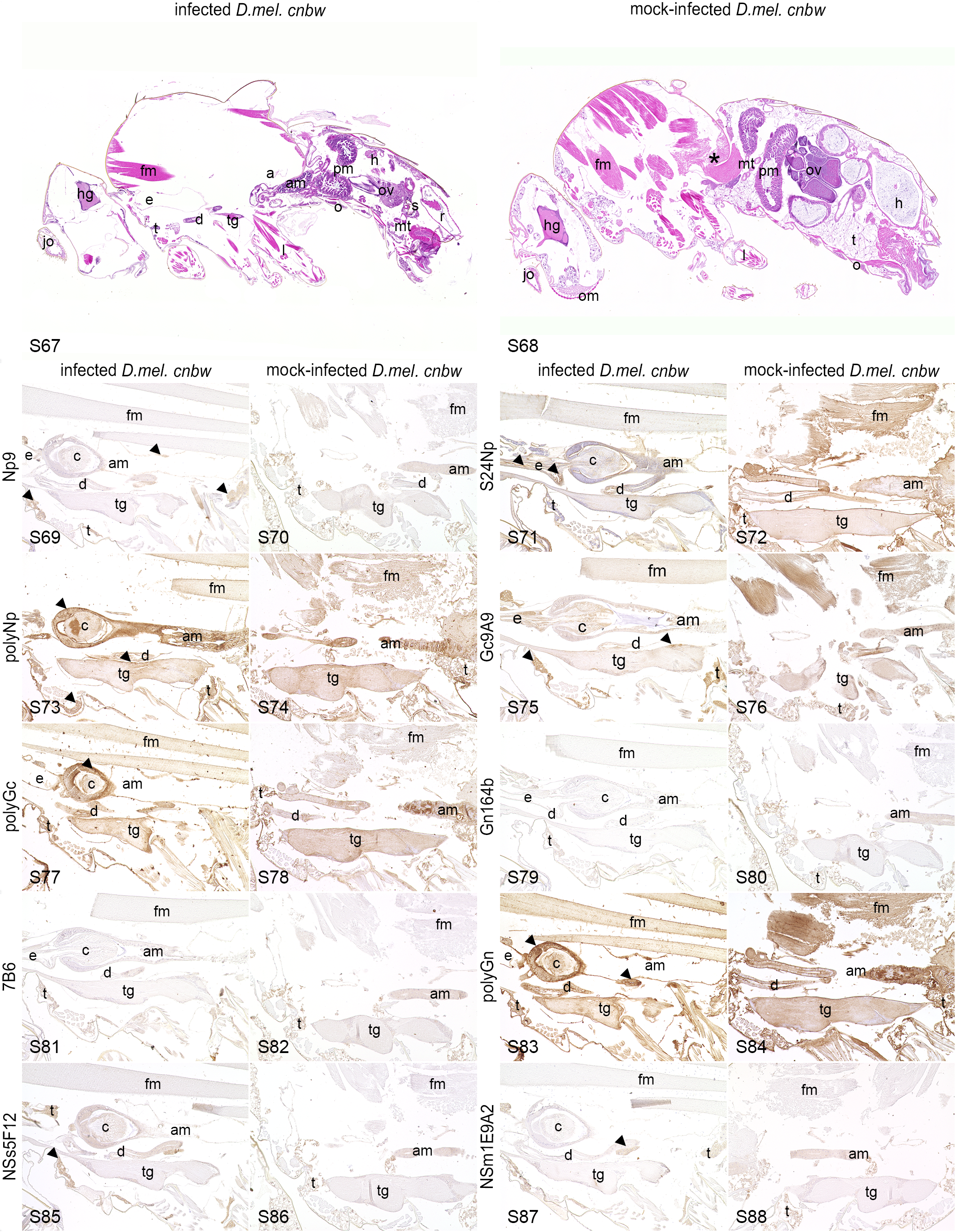

Supplement: Supplementary file 5 — Supplementary Information 5. [file 41598_2021_89226_MOESM5_ESM.tif]

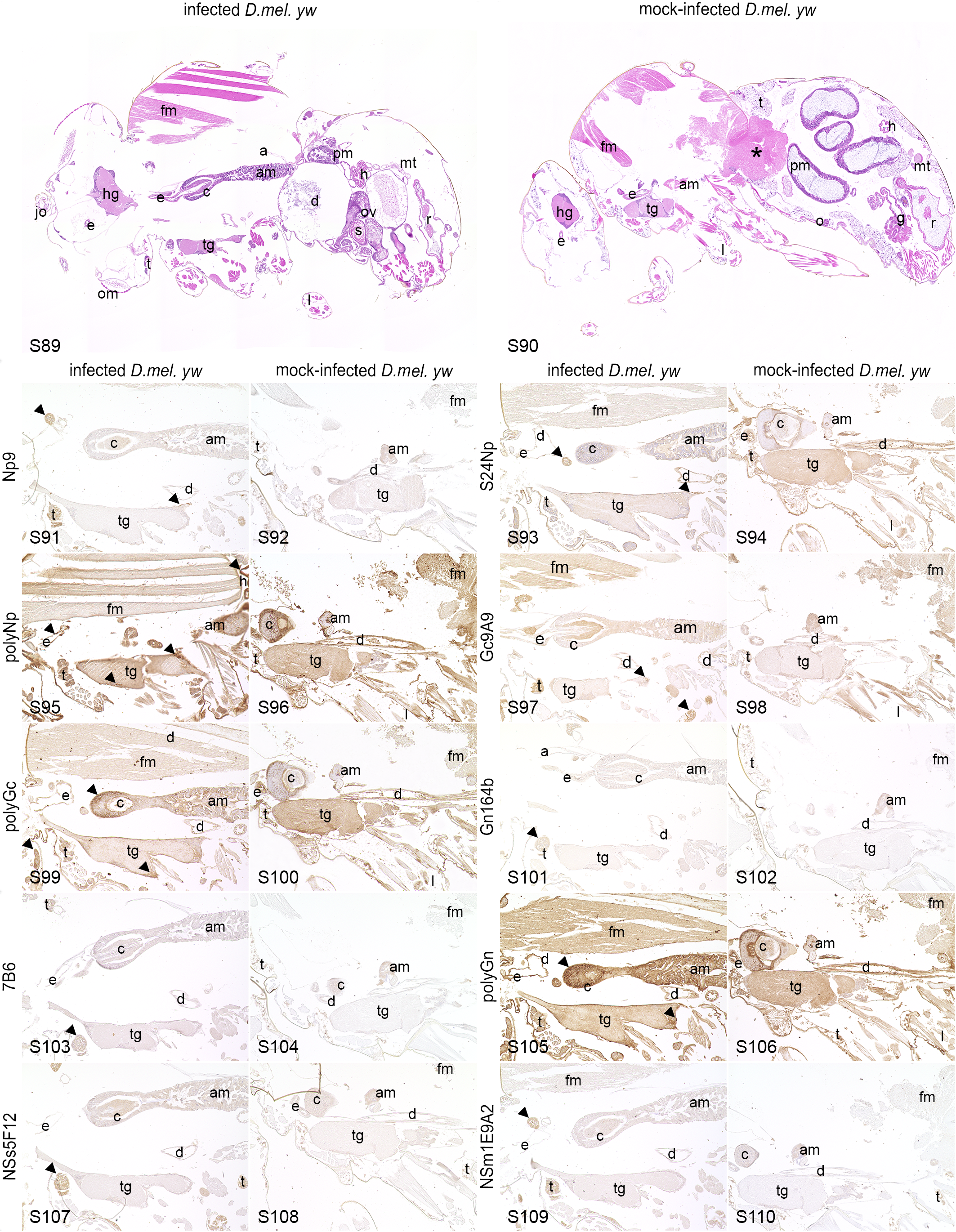

Supplement: Supplementary file 6 — Supplementary Information 6. [file 41598_2021_89226_MOESM6_ESM.tif]

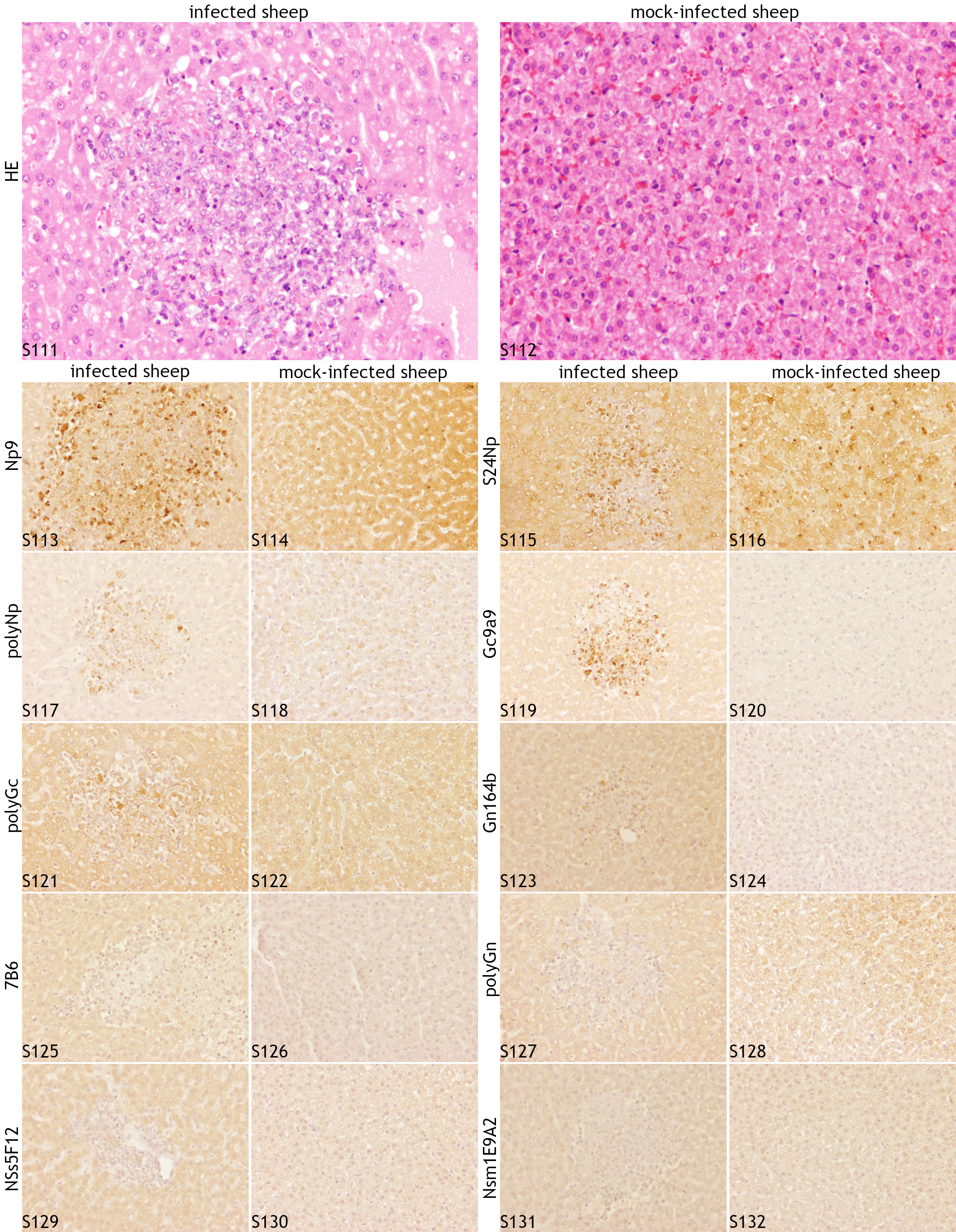

Supplement: Supplementary file 7 — Supplementary Information 7. [file 41598_2021_89226_MOESM7_ESM.tif]

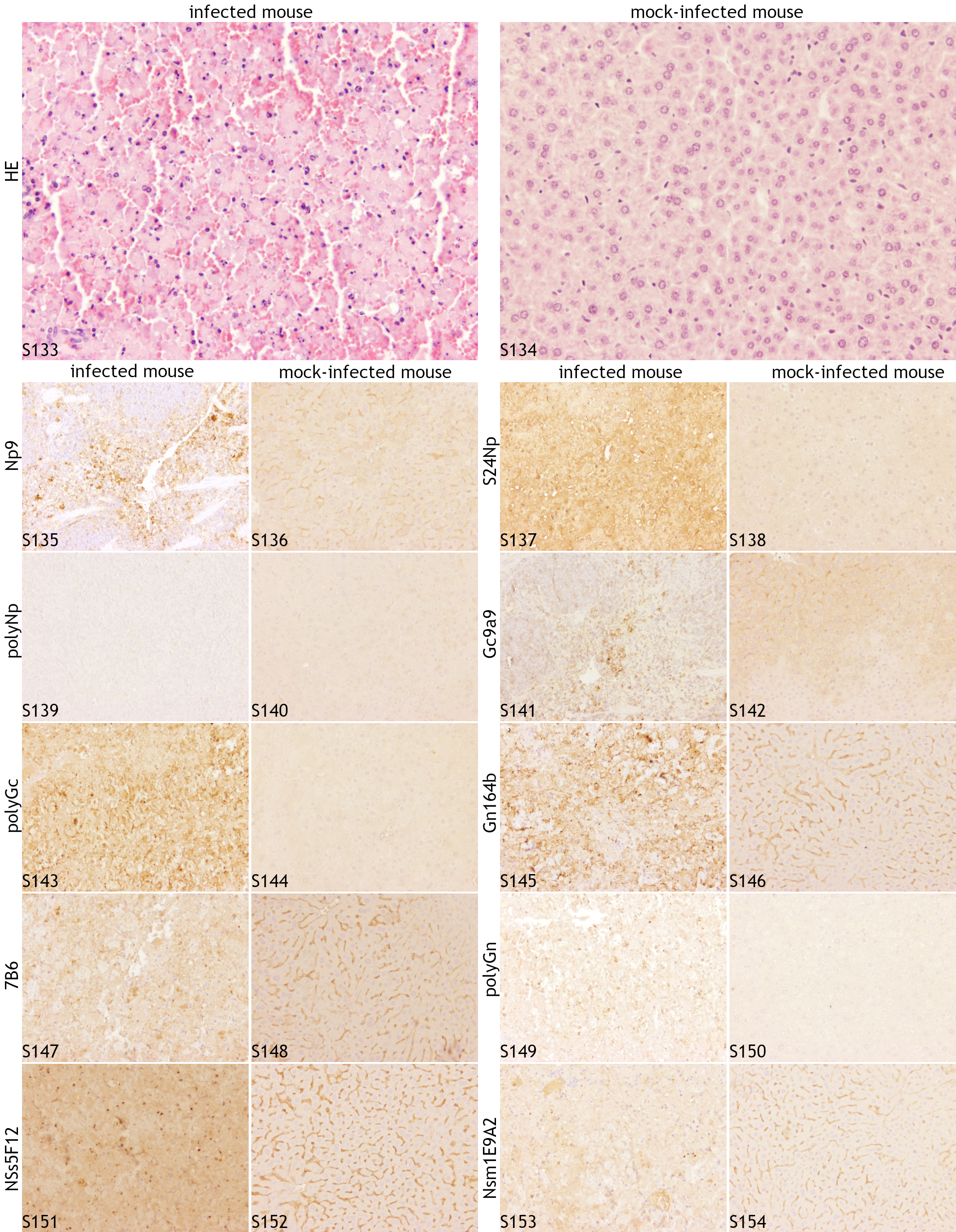

Supplement: Supplementary file 8 — Supplementary Information 8. [file 41598_2021_89226_MOESM8_ESM.tif]

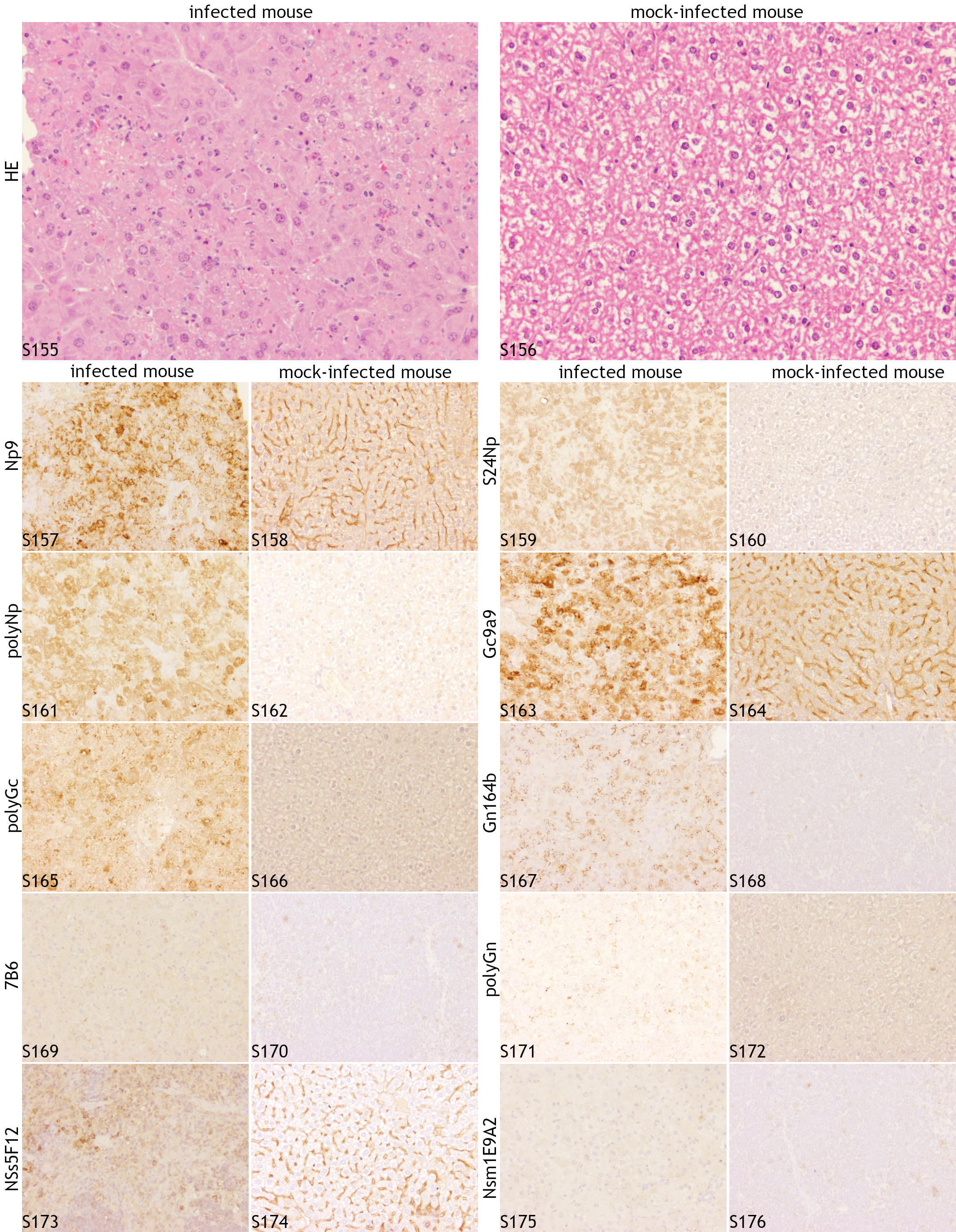

Supplement: Supplementary file 9 — Supplementary Information 9. [file 41598_2021_89226_MOESM9_ESM.tif]
